# Does Long-Term Post-Bariatric Weight Change Differ Across Antidepressants?

**DOI:** 10.1097/AS9.0000000000000114

**Published:** 2022-01-10

**Authors:** David E. Arterburn, Matthew L. Maciejewski, Theodore S. Z. Berkowitz, Valerie A. Smith, James E. Mitchell, Chuan-Fen Liu, Adenike Adeyemo, Katharine A. Bradley, Maren K. Olsen

**Affiliations:** From the * Kaiser Permanente Washington Health Research Institute, Seattle, WA; † Department of Medicine, University of Washington, Seattle, WA; ‡ Center of Innovation to Accelerate Discovery and Practice Transformation, Durham VA Medical Center, Durham, NC; § Department of Population Health Sciences, Duke University, Durham, NC; ∥ Division of General Internal Medicine, Department of Medicine, Duke University, Durham, NC; ¶ University of North Dakota School of Medicine and Health Sciences, Fargo, ND; # Department of Health Services, University of Washington, Seattle, WA; ** Department of Biostatistics and Bioinformatics, Duke University, Durham, NC.

**Keywords:** antidepressants, bariatric, bupropion, gastric bypass, matching, sleeve gastrectomy, veterans, weight

## Abstract

**Objectives:**

We sought to evaluate whether weight change up to 5 years after bariatric surgery differed by antidepressant class taken before surgery.

**Background:**

Bariatric surgery induces significant weight loss, but outcomes are highly variable. The specific type of antidepressant used prior to surgery may be an important factor in long-term weight loss.

**Methods:**

This retrospective cohort study from 2000 to 2016 compared the 5-year weight loss of 556 Veterans who were taking antidepressant monotherapy (bupropion, selective serotonin reuptake inhibitors [SSRIs], or serotonin-norepinephrine reuptake inhibitors [SNRIs]) before bariatric surgery (229 sleeve gastrectomy and 327 Roux-en-Y gastric bypass) versus 556 matched nonsurgical controls.

**Results:**

Patients taking bupropion before sleeve gastrectomy had greater differential weight loss between surgical patients and matched controls than those taking SSRIs at 1 (8.9 pounds; 95% confidence interval [CI], 1.6–16.3; *P* = 0.02) and 2 years (17.6 pounds; 95% CI, 5.9–29.3; *P* = 0.003), but there was no difference at 5 years (11.9 pounds; 95% CI, –8.9 to 32.8; *P* = 0.26). Findings were similar for gastric bypass patients taking bupropion compared to SSRIs at 1 (9.7 pounds; 95% CI, 2.0–17.4; *P* = 0.014), 2 (12.0 pounds; 95% CI, –0.5 to 24.5; *P* = 0.06), and 5 years (4.8 pounds; 95% CI, –16.7 to 26.3; *P* = 0.66). No significant differences were observed comparing patients taking SNRI versus SSRI medications.

**Conclusions:**

Sleeve gastrectomy and gastric bypass patients taking bupropion had greater weight loss than those taking SSRIs, although these differences may wane over time. Bupropion may be the first-line antidepressant of choice among patients with severe obesity considering bariatric surgery.

## Abstract 2

Mini-abstract: Bariatric surgery induces significant weight loss, but outcomes are highly variable. We found that sleeve gastrectomy and gastric bypass patients taking bupropion had greater weight loss than those taking selective serotonin reuptake inhibitors, although these differences may wane over time. Bupropion may be the first-line antidepressant of choice among patients considering bariatric surgery.

Supplemental Digital Content is available in the text.

## INTRODUCTION

Bariatric surgery is currently the most effective intervention for inducing long-term weight loss among patients with severe obesity.^1^ However, the long-term weight trajectories of postsurgical patients vary widely,^2^ and there is a clear need for research that identifies modifiable preoperative clinical factors that can maximize long-term weight loss.

Depression is highly prevalent among patients who undergo bariatric surgery, with preoperative prevalence estimates ranging from 35% to 44%.^3,4^ Evidence is mixed regarding the impact of depression on weight loss after bariatric surgery, with some research suggesting no effect and other studies finding reductions in weight loss.^3–6^ This literature is difficult to synthesize, owing to many studies with short durations of follow-up and wide variations in the types of antidepressant medications evaluated. Another consideration is the finding that bariatric surgery appears to decrease the absorption of some antidepressants,^7^ which might change their clinical effectiveness as well as alter their effects on weight loss.^5–7^

Different classes of antidepressant medications are known to have a clinically meaningful impact on long-term weight trajectories in general populations of adults with depression.^8^ Among the most commonly prescribed antidepressant medications, the norepinephrine and dopamine reuptake inhibitor (NDRI) medication, bupropion, is associated with weight loss, while selective serotonin reuptake inhibitors (SSRIs) and serotonin-norepinephrine reuptake inhibitors (SNRIs) have been largely found to be weight neutral or promote small amounts of weight gain. Given the high prevalence of depression among patients undergoing bariatric surgery, it could be clinically helpful to know if the preoperative choice of antidepressant medication has a significant impact on long-term surgical weight loss outcomes.

A few prior studies have attempted to identify whether antidepressant use is associated with weight change in surgical patients.^9–12^ Two studies found that patients taking antidepressants before surgery had similar weight loss at 1 year compared to patients not taking antidepressants,^9,12^ but 1 study found that patients continuing antidepressants after surgery lost less weight at 24 months than patients not continuing antidepressants.^11^ Patients taking SNRIs lost more weight than patients taking SSRIs in 1 study,^12^ but lost less weight than patients not taking antidepressants after surgery in another study.^11^ These studies tended to only examine follow-up up to 1 year, focused on a single surgical procedure (most often gastric bypass) from a single site, and did not contrast weight change of surgical and matched nonsurgical control patients.

To address these gaps, we conducted a retrospective observational study involving a multisite cohort of Veterans with depression undergoing bariatric surgery matched to nonsurgical controls. The purpose was to examine whether postsurgical weight change differed between surgical patients and nonsurgical controls on 3 classes of antidepressants. We compared patients who were prescribed bupropion and SNRIs before surgery to those taking the more commonly prescribed SSRIs at baseline. We hypothesized that bupropion use at baseline would be associated with more weight loss than SSRIs and that SSRI and SNRI medications would have similar weight loss.

## METHODS

### Study Design and Study Population

In this retrospective cohort study based on data from Veterans Affair’s (VA’s) Corporate Data Warehouse that aggregates electronic health record data from all VA medical centers nightly, we initially identified 10,653 Veterans who underwent a bariatric operation at any VA bariatric center between October 1, 2000, and September 30, 2016. The Institutional Review Boards of the Durham VA and Kaiser Permanente Washington Health Research Institute approved this study. We excluded those patients who had reoperations, did not have documented obesity, had preoperative cancer, had other medical exclusions, received uncommon bariatric procedures, and patients who either (1) did not have depression, (2) were not receiving antidepressant treatment, or (3) or were receiving multiple antidepressant medications at the time of surgery (eFigure 1, http://links.lww.com/AOSO/A89). The final surgical cohort included 229 patients who underwent a laparoscopic sleeve gastrectomy and 327 patients who underwent a Roux-en-Y gastric bypass operation who were receiving antidepressant monotherapy at the time of surgery. The cohort was stratified based on antidepressant class into those taking only 1 of 3 antidepressant classes: SSRI, SNRI, or NDRI only. Antidepressant use was defined as having 2 or more fills of 30+ days duration in the 180 days prior to their surgery date (see eTable 1, http://links.lww.com/AOSO/A89 for specific medications included). Medication data was extracted from VA’s Pharmacy Benefits Management databases.

These surgical patients were matched 1-to-1 to comparable nonsurgical controls using sequential stratification matching to accommodate the time-varying nature of surgical eligibility characteristics (eg, body mass index [BMI]) after being identified from the VA electronic health record.^13–15^ Data were organized into a series of n-of-1 randomized clinical trials, in which the trial start date was the surgical date of each patient. Matching was conducted within each of the antidepressant classes. For example, for each surgical patient with SSRI monotherapy, we identified a pool of eligible potential control patients with SSRI monotherapy who had not yet had a bariatric surgical procedure but had similar binary characteristics associated with the outcomes and the likelihood of receiving a surgical procedure (eg, sex, race/ethnicity [White or non-White], diabetes diagnosis [all-time lookback], chronic prescription opioid use, unhealthy alcohol use [2-year lookback], and alcohol use disorder [2-year lookback] documented in the VA databases). Surgical patients and potential control patients were required to be within 5 years of age and have similar baseline BMI measurements (calculated as weight in kilograms divided by height in meters squared) within 6 months before the surgical date. Surgical patients without a representative match were excluded (eFigure 1, http://links.lww.com/AOSO/A89). The majority (n = 401 of 556) of surgical patients had more than 1 eligible match. Among those with more than 1 potential match, the match with most weight measurements during the study period was chosen as the best match. The final control cohort included 229 patients who matched to 229 laparoscopic sleeve gastrectomy patients and 327 patients who matched to 327 Roux-en-Y gastric bypass patients.

### Weight Change Outcome

Follow-up weight data up to 6 years after the index date were obtained from measurements recorded in the electronic health record during outpatient visits between October 1, 1999, and March 31, 2020. For each individual surgical patient and match, the analytic model included all valid weights measured from 1 year prior to the surgical patient’s date of surgery through 5 years after surgery. The primary comparisons of interest were the difference in change in weight between surgical and nonsurgical patients from baseline (index or surgery) to 1, 2, and 5 years after surgery, comparing patients taking bupropion and SSRIs at baseline and those taking SNRIs and SSRIs at baseline, separately.

### Statistical Analysis

Descriptive summary statistics and individual trajectory plots and penalized B-spline plots by antidepressant class and surgery type were used to guide initial model specification. As expected, surgical patients’ weights decreased after surgery, with a leveling off between 6 and 18 months postsurgery, depending upon the type of surgery. Weight trajectories among the matched nonsurgical controls showed little change over time.

Potential mean model specification included quadratic, cubic, quartic, and parametric piece-wise linear splines, with separately specified random effects for cases and matches. Final model selection was guided by lowest Akaike information criterion indices. Our final model included separate intercepts for each antidepressant class within surgical cases and matches, linear time, linear time interactions for each antidepressant class, linear piece-wise splines at surgery, 6 months, 1, 2, 3, and 4 years for the surgical cases, and interactions of the piece-wise splines with antidepressant class. Random effect specification included intercepts, linear, and quadratic time, grouped by surgical case indicator. This model specification resulted in 6 estimated mean trajectories over the 5-year study period—one for each antidepressant class, by surgical case and match and was fit with PROC MIXED in SAS v9.4 (Cary, NC). Estimate statements were used to generate estimated change in weights in pounds at 1, 2, and 5 years of follow-up within each antidepressant class and case or match subgroup and to estimate comparisons of cases and matches over time between the antidepressant classes.

## RESULTS

### Descriptive Statistics

Patients undergoing sleeve gastrectomy and controls taking antidepressants before surgery were well balanced on the covariates used for matching (top of Table 1), with an average age of 52–53 years, average BMI of 42.4–43.6 kg/m^2^ and 25%–39% female. The SSRIs were more commonly taken at baseline (n = 162 sleeve patients) than either SNRIs (n = 39 sleeve patients) or NDRIs (n = 28 sleeve patients). There were several covariates (eg, marital status, diagnosed anxiety, diagnosed post-traumatic stress disorder) not used in matching that were imbalanced (bottom of Table 1). A smaller proportion of patients taking bupropion were of White race or had diagnosed diabetes than patients taking SNRIs or SSRIs.

**TABLE 1.** Baseline Characteristics of Sleeve Gastrectomy and Matched Controls, by Antidepressant Class

Variables	NDRIs Only		SNRIs Only		SSRIs Only	
	Sleeve (n = 28)	Controls (n = 28)	Sleeve (n = 39)	Controls (n = 39)	Sleeve (n = 162)	Controls (n = 162)
Variables used in match						
Female, N (%)	11 (39.3)	11 (39.3)	10 (25.6)	10 (25.6)	42 (25.9)	42 (25.9)
Age, mean (SD)	52.9 (8.2)	53.4 (8.4)	51.9 (10.5)	53.0 (10.5)	53.9 (9.8)	53.9 (10.0)
BMI, mean (SD)	43.6 (5.6)	42.7 (5.2)	42.7 (5.0)	42.4 (5.0)	43.3 (5.9)	42.9 (5.4)
Race, White, N (%)	21 (75.0)	21 (75.0)	35 (89.7)	35 (89.7)	142 (87.7)	142 (87.7)
Diagnosed diabetes, N (%)*	10 (35.7)	10 (35.7)	23 (59.0)	23 (59.0)	92 (56.8)	92 (56.8)
Alcohol misuse, N (%)	0 (0.0)	0 (0.0)	1 (2.6)	1 (2.6)	18 (11.1)	18 (11.1)
Chronic prescription opioid use, N (%)	6 (21.4)	6 (21.4)	18 (46.2)	18 (46.2)	57 (35.2)	57 (35.2)
Diagnosed alcohol use disorder, N (%)*	1 (3.6)	1 (3.6)	1 (2.6)	1 (2.6)	17 (10.5)	17 (10.5)
Diagnosed opioid use disorder, N (%)*	0 (0.0)	0 (0.0)	0 (0.0)	0 (0.0)	0 (0.0)	0 (0.0)
Variables not used in match						
DCG risk score, mean (SD)	NA (NA)	NA (NA)	NA (NA)	NA (NA)	0.3 (NA)	0.9 (NA)
Nosos risk score, mean (SD)	1.8 (1.1)	1.9 (1.0)	1.9 (1.0)	1.8 (1.0)	2.0 (1.1)	1.8 (1.1)
Married, N (%)	16 (57.1)	12 (42.9)	24 (61.5)	15 (38.5)	85 (52.5)	65 (40.1)
Previously married, N (%)	7 (25.0)	9 (32.1)	11 (28.2)	12 (30.8)	58 (35.8)	53 (32.7)
Unmarried or unknown, N (%)	5 (17.9)	7 (25.0)	4 (10.3)	12 (30.8)	19 (11.7)	44 (27.2)
VA outpatient mental health visits, mean (SD)	11.6 (9.3)	18.0 (29.6)	18.6 (13.9)	14.5 (13.6)	14.6 (17.6)	14.0 (21.5)
Diagnosed PTSD, N (%)*	6 (21.4)	12 (42.9)	19 (48.7)	14 (35.9)	58 (35.8)	57 (35.2)
Diagnosed cannabis disorder, N (%)*	0 (0.0)	0 (0.0)	0 (0.0)	3 (7.7)	1 (0.6)	3 (1.9)
Diagnosed other drug disorder, N (%)*	2 (7.1)	2 (7.1)	0 (0.0)	1 (2.6)	7 (4.3)	4 (2.5)
Diagnosed anxiety, N (%)*	8 (28.6)	9 (32.1)	11 (28.2)	15 (38.5)	44 (27.2)	37 (22.8)
Diagnosed bipolar, N (%)*	2 (7.1)	3 (10.7)	2 (5.1)	2 (5.1)	9 (5.6)	13 (8.0)
Diagnosed psychosis, N (%)*	0 (0.0)	0 (0.0)	0 (0.0)	1 (2.6)	2 (1.2)	2 (1.2)
Diagnosed schizophrenia, N (%)*	1 (3.6)	1 (3.6)	0 (0.0)	0 (0.0)	2 (1.2)	3 (1.9)
Diagnosed eating disorder, N (%)*	3 (10.7)	0 (0.0)	0 (0.0)	0 (0.0)	2 (1.2)	1 (0.6)
Diagnosed tobacco use disorder, N (%)*	3 (10.7)	4 (14.3)	6 (15.4)	7 (17.9)	19 (11.7)	23 (14.2)
Diagnosed GERD, N (%)*	13 (46.4)	10 (35.7)	14 (35.9)	9 (23.1)	48 (29.6)	39 (24.1)

*All diagnoses were identified from inpatient and outpatient visit records using International Classification of Diseases-9 and International Classification of Diseases-10 codes.

DCGs indicates diagnostic cost groups; GERD, gastroesophageal reflux disorder; NA, not available; PTSD, post-traumatic stress disorder.

Patients undergoing gastric bypass and matched controls taking antidepressants before surgery were also well matched on the covariates used for matching (top of Table 2), with an average age of 51–53 years, average BMI of 41.8–46.4 kg/m^2^ and 28%–34% female. The SSRIs were more commonly taken at baseline (n = 266 gastric bypass patients) than either SNRIs (n = 32 gastric bypass patients) or NDRIs (n = 29 gastric bypass patients). Again, there were several covariates (eg, marital status, diagnosed post-traumatic stress disorder, smoking status) not used in matching that were imbalanced (bottom of Table 2). Both the sleeve and gastric bypass patients and matched controls had similar numbers of weight observations and rates of follow-up among the antidepressant therapy classes (eTable 2, http://links.lww.com/AOSO/A89).

**TABLE 2.** Baseline Characteristics of Gastric Bypass and Matched Controls, by Antidepressant Class

Variables	NDRIs Only		SNRIs Only		SSRIs Only	
	RYGB (n = 29)	Controls (n = 29)	RYGB (n = 32)	Controls (n = 32)	RYGB (n = 266)	Controls (n = 266)
Variables used in match						
Female, N (%)	8 (27.6)	8 (27.6)	11 (34.4)	11 (34.4)	83 (31.2)	83 (31.2)
Age, mean (SD)	50.9 (6.6)	51.3 (8.0)	51.2 (8.3)	51.4 (8.1)	53.0 (8.0)	52.9 (8.3)
BMI, mean (SD)	44.1 (6.0)	41.8 (4.3)	45.0 (6.2)	43.8 (5.5)	46.4 (7.2)	45.3 (6.5)
Race, White, N (%)	22 (75.9)	22 (75.9)	28 (87.5)	28 (87.5)	227 (85.3)	227 (85.3)
Diagnosed diabetes, N (%)*	17 (58.6)	17 (58.6)	19 (59.4)	19 (59.4)	155 (58.3)	155 (58.3)
Alcohol misuse, N (%)	0 (0.0)	0 (0.0)	1 (3.1)	1 (3.1)	7 (2.6)	7 (2.6)
Chronic prescription opioid use, N (%)	13 (44.8)	13 (44.8)	14 (43.8)	14 (43.8)	91 (34.2)	91 (34.2)
Diagnosed alcohol use disorder, N (%)*	2 (6.9)	2 (6.9)	2 (6.3)	2 (6.3)	20 (7.5)	20 (7.5)
Diagnosed opioid use disorder, N (%)*	0 (0.0)	0 (0.0)	1 (3.1)	1 (3.1)	1 (0.4)	1 (0.4)
Variables not used in match						
DCG risk score, mean (SD)	0.7 (0.5)	0.6 (0.4)	0.5 (0.3)	0.4 (0.1)	0.8 (0.6)	0.9 (0.6)
Nosos risk score, mean (SD)	2.0 (0.7)	2.2 (1.6)	1.9 (0.8)	1.8 (0.9)	1.9 (1.0)	1.9 (1.3)
Married, N (%)	10 (34.5)	12 (41.4)	14 (43.8)	13 (40.6)	150 (56.4)	116 (43.6)
Previously married, N (%)	15 (51.7)	10 (34.5)	13 (40.6)	12 (37.5)	80 (30.1)	67 (25.2)
Unmarried or unknown, N (%)	4 (13.8)	7 (24.1)	5 (15.6)	7 (21.9)	36 (13.5)	83 (31.2)
VA outpatient mental health visits, mean (SD)	15.8 (19.9)	21.3 (26.8)	13.9 (14.1)	12.6 (11.7)	12.3 (16.8)	11.6 (24.6)
Diagnosed PTSD, N (%)*	7 (24.1)	7 (24.1)	12 (37.5)	16 (50.0)	86 (32.3)	70 (26.3)
Diagnosed cannabis disorder, N (%)*	0 (0.0)	0 (0.0)	0 (0.0)	0 (0.0)	4 (1.5)	5 (1.9)
Diagnosed other drug disorder, N (%)*	1 (3.4)	2 (6.9)	2 (6.3)	1 (3.1)	9 (3.4)	11 (4.1)
Diagnosed anxiety, N (%)*	8 (27.6)	8 (27.6)	7 (21.9)	12 (37.5)	51 (19.2)	55 (20.7)
Diagnosed bipolar, N (%)*	5 (17.2)	2 (6.9)	2 (6.3)	4 (12.5)	19 (7.1)	12 (4.5)
Diagnosed psychosis, N (%)*	0 (0.0)	1 (3.4)	1 (3.1)	0 (0.0)	2 (0.8)	4 (1.5)
Diagnosed schizophrenia, N (%)*	0 (0.0)	2 (6.9)	0 (0.0)	0 (0.0)	1 (0.4)	18 (6.8)
Diagnosed eating disorder, N (%)*	0 (0.0)	1 (3.4)	1 (3.1)	1 (3.1)	7 (2.6)	0 (0.0)
Diagnosed tobacco use disorder, N (%)*	3 (10.3)	6 (20.7)	3 (9.4)	5 (15.6)	20 (7.5)	39 (14.7)
Diagnosed GERD, N (%)*	7 (24.1)	10 (34.5)	13 (40.6)	8 (25.0)	72 (27.1)	61 (22.9)

*All diagnoses were identified from inpatient and outpatient visit records using International Classification of Diseases-9 and International Classification of Diseases-10 codes.

DCGs indicates diagnostic cost groups; GERD, gastroesophageal reflux disorder; PTSD, post-traumatic stress disorder; RYGB, Roux-en-Y gastric bypass.

### Differences in Weight Change by Antidepressant Monotherapy in Sleeve Gastrectomy Cohort

**FIGURE 1.**
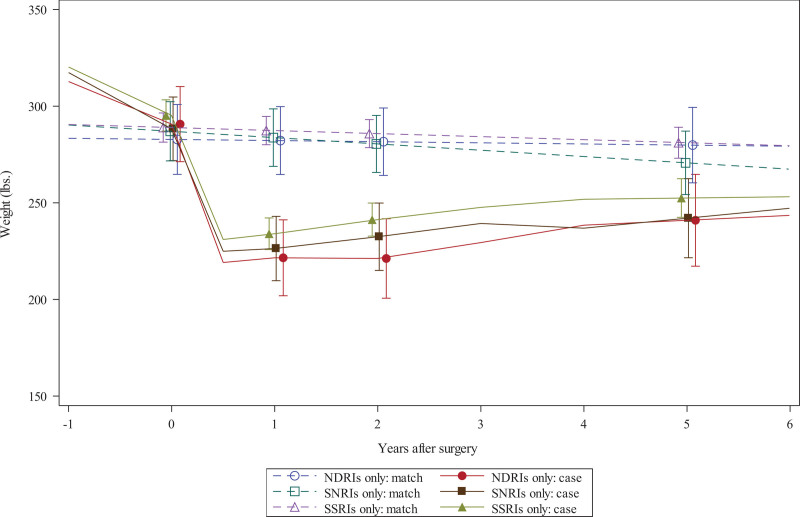
Estimated changes in weight over time among sleeve gastrectomy patients and nonsurgical matches taking 1 of 3 antidepressants before surgery. lbs indicates pounds.

In the sleeve gastrectomy cohort, mean weights of surgical and nonsurgical patients were similar at baseline (Fig. 1), but surgical patients’ mean weight change 1 year after surgery was 59–69 pounds lower than matched controls (depending on their antidepressant used at baseline; see top of Table 3). Compared with patients taking SSRIs at baseline, patients taking NDRIs at baseline had greater differential weight loss between surgical patients and matched controls at 1 year (8.9 pounds; 95% confidence interval [CI], 1.6–16.3; *P* = 0.02; Table 3).

**TABLE 3.** Estimated Surgical Effect on Weight Change From Baseline and Difference in Surgical Effect Between SSRI and Other Antidepressant Classes

Treatment Groups	Time Since Bariatric Surgery								
	Weight Change at 1 y From Baseline			Weight Change at 2 y From Baseline			Weight Change at 5 y From Baseline		
	Surgical Weight (95% CI)	Control Weight (95% CI)	Diff Surg-Control	Surgical Weight (95% CI)	Control Weight (95% CI)	Diff Surg-Control	Surgical Weight (95% CI)	Control Weight (95% CI)	Diff Surg-Control
Sleeve gastrectomy cohort									
SSRI	–61.2 (–63.8 to –58.6)	–1.6 (–2.7 to –0.5)	–59.6 (–62.5 to –56.8)	–53.9 (–57.8 to –50.0)	–3.1 (–5.3 to –0.9)	–50.8 (–55.2 to –46.3)	–42.7 (–48.5 to –36.9)	–7.8 (–13.3 to –2.4)	–34.9 (–42.8 to –26.9)
SNRI	–61.9 (–67.2 to –56.6)	–3.3 (–5.5 to –1.0)	–58.7 (–64.4 to –52.9)	–55.8 (–63.8 to –47.9)	–6.5 (–11.1 to –2.0)	–49.3 (–58.5 to –40.1)	–46.2 (–58.3 to –34.2)	–16.3 (–27.6 to –5.0)	–29.9 (–46.4 to –13.4)
NDRI	–69.2 (–75.3 to –63.0)	–0.6 (–3.3 to 2.1)	–68.6 (–75.3 to –61.8)	–69.5 (–78.8 to –60.2)	–1.2 (–6.6 to 4.3)	–68.3 (–79.1 to –57.6)	–49.7 (–63.3 to –36.2)	–2.9 (–16.6 to 10.7)	–46.8 (–66.0 to –27.6)
Diff_SSRI–SNRI_			–1.0 (–7.4 to 5.4; *P* = 0.77)			–1.4 (–11.7 to 8.8; *P* = 0.78)			–4.9 (–23.3 to 13.4; *P* = 0.60)
Diff_SSRI–NDRI_			8.9 (1.6–16.3; *P* = 0.016)			17.6 (5.9–29.3; *P* = 0.003)			11.9 (–8.9 to 32.8; *P* = 0.26)
Gastric bypass cohort									
SSRI	–88.3 (–90.5 to –86.0)	–1.3 (–2.2 to –0.3)	–87.0 (–89.4 to –84.5)	–87.0 (–90.5 to –83.6)	–2.6 (–4.5 to –0.7)	–84.4 (–88.4 to –80.5)	–76.3 (–81.0 to –71.5)	–6.4 (–11.2 to –1.7)	–69.8 (–76.6 to –63.1)
SNRI	–90.0 (–96.5 to –83.4)	–2.8 (–5.6 to –0.1)	–87.1 (–94.2 to –80.1)	–84.8 (–94.8 to –74.7)	–5.6 (–11.1 to –0.2)	–79.1 (–90.6 to –67.7)	–81.7 (–95.8 to –67.5)	–14.1 (–27.8 to –0.4)	–67.6 (–87.3 to –47.9)
NDRI	–96.9 (–103.7 to –90.2)	–0.3 (–3.2 to 2.6)	–96.7 (–104.0 to –89.3)	–97.0 (–107.4 to –86.6)	–0.6 (–6.4 to 5.2)	–96.4 (–108.3 to –84.5)	–76.1 (–90.4 to –61.8)	–1.5 (–16.0 to 13.1)	–74.7 (–95.1 to –54.3)
Diff_SSRI–SNRI_			0.2 (–7.3 to 7.6; *P* = 0.96)			–5.3 (–17.4 to 6.8; *P* = 0.39)			–2.3 (–23.1 to 18.6; *P* = 0.83)
Diff_SSRI–NDRI_			9.7 (2.0–17.4; *P* = 0.014)			12.0 (–0.6 to 24.5; *P* = 0.06)			4.8 (–16.7 to 26.3; *P* = 0.66)

Sleeve gastrectomy patients’ weight 2 years after surgery was 49–68 pounds lower than matched controls (depending on antidepressant). The relative improvement in weight loss among sleeve gastrectomy patients compared with matched controls was again greater for those taking NDRIs at baseline compared with those taking SSRIs (17.6 pounds; 95% CI, 5.9–29.3; *P* = 0.003).

Five years after surgery, sleeve gastrectomy patients’ weight change was 30–47 pounds lower than matched controls (depending on antidepressant), but there were no statistically significant differences in weight loss between surgical and matched nonsurgical patients comparing those taking NDRI and those taking SSRIs at baseline (–4.9 pounds for SSRI vs SNRI [95% CI, –23.3 to 13.4; *P* = 0.60] and 11.9 pounds for NDRI vs SSRI [95% CI, –8.9 to 32.8; *P* = 0.26]).

Finally, comparing patients taking SSRI and SNRIs, there were no significant differences between sleeve gastrectomy patients and matched controls in 1-, 2-, or 5-year weight loss outcomes.

### Differences in Weight Change by Antidepressant Monotherapy in Gastric Bypass Cohort

**FIGURE 2.**
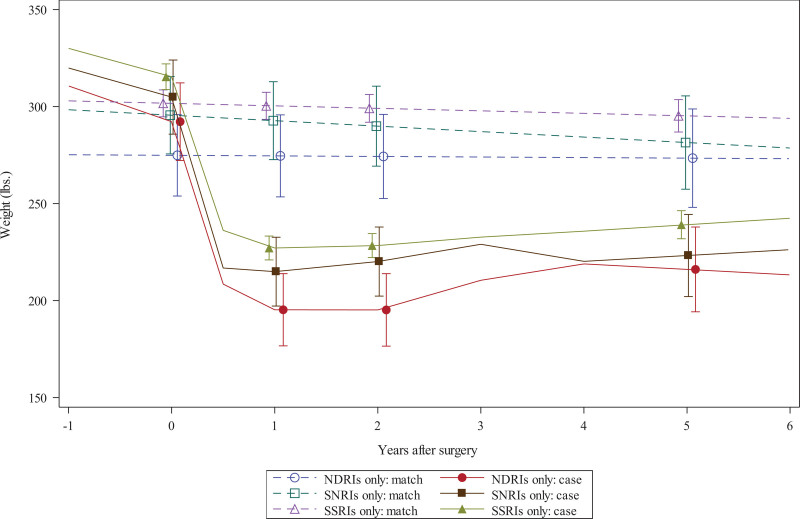
Estimated changes in weight over time among gastric bypass patients and nonsurgical matches taking 1 of 3 antidepressants before surgery. lbs indicates pounds.

In the gastric bypass cohort, mean weights of surgical and nonsurgical patients were similar at baseline for patients taking SSRIs or NDRIs (Fig. 2). Surgical patients’ change in weight from baseline to 1 year after surgery was 87 to 97 pounds lower than matched controls (depending on antidepressant; see bottom of Table 3). Compared with patients taking SSRIs at baseline, those taking NDRIs had greater differential weight loss between surgical patients and matched controls at 1 year (9.7 pounds; 95% CI, 2.0–17.4; *P* = 0.014; Table 3).

Gastric bypass patients’ change in weight from baseline to 2 years after surgery was 79–96 pounds lower than matched controls (depending on antidepressant; Table 3), and compared with patients taking SSRIs at baseline, differential weight loss at 2 years between surgical patients and matched controls was 12.0 pounds greater for the NDRI cohort (95% CI, –0.5 to 24.5; *P* = 0.06; Table 3), although this difference was not statistically significant. Five years after surgery, gastric bypass patients’ change in weight from baseline was 68–75 pounds lower than matched controls (depending on antidepressant) and, by 5 years, surgical-matched weight improvements were not significantly different across patients taking NDRIs versus SSRIs (4.8 pounds; 95% CI, –16.7 to 26.3; *P* = 0.66; Table 3).

There were no significant differences in 1-, 2-, or 5-year weight loss outcomes comparing gastric bypass patients and matched control taking SSRIs versus those taking SNRIs at baseline.

## COMMENT

This study tested the association between taking 3 classes of antidepressant medications prior to bariatric surgery and weight loss in the 5 years after surgery. Specifically, the study compared weight loss in those undergoing bariatric surgery and matched nonsurgical controls in patients taking bupropion, SNRIs, or SSRIs at baseline. We found that patients taking bupropion (a NDRI medication) had greater weight loss compared with nonsurgical controls than patients taking SSRIs at 1 and 2 years after surgery. However, differences resolved by 5 years. Those taking SNRIs had similar weight loss as those taking SSRIs. Our findings were similar for patients who had sleeve gastrectomy and gastric bypass surgery. Taken as a whole, this study suggests that the choice of antidepressant medication prior to bariatric surgery may have an important impact on weight loss outcomes. This finding is clinically relevant because as many as one-third to one-half of bariatric patients have a history of major depression at baseline, and preoperative antidepressant treatment is common.^3,4^

This study improves upon prior studies that had limited follow-up to just 12^9,12^ or 24 months^11^ after surgery and examined only sleeve gastrectomy or gastric bypass. In addition, we compared the weight change of surgical patients taking each class of antidepressants with matched nonsurgical controls taking the same antidepressant class to have a counterfactual weight trend in contrast to the trends for each antidepressant class; none of the prior studies included nonsurgical controls. This design difference is important because our findings also suggest that bariatric surgery is more effective in promoting long-term weight loss compared than usual care regardless of the use of antidepressant medications.

Antidepressant medications are widely believed to have similar efficacy for the treatment of depression.^16^ Thus, the decision about which antidepressant medication to use often revolves around the expected side effects of the medications, their costs, and patient preferences.^17^ Prior research has demonstrated that, for adults, initiation of bupropion was associated with 7.1 pounds greater weight loss at 2 years (95% CI, –11.3 to –2.8; *P* < 0.01) than people who initiated fluoxetine (a SSRI).^18^ The current study extends these findings to patients taking bupropion at the time of bariatric surgery. Given that bupropion is the only antidepressant associated with long-term weight loss, these studies suggest that bupropion should be the first-line drug of choice for all overweight and obese patients—including those seeking bariatric surgery—unless there are other existing contraindications (eg, history of seizure disorder, anorexia nervosa or bulimia, or patients undergoing abrupt discontinuation of ethanol or sedatives including anticonvulsants, barbiturates, or benzodiazepines).

Several factors likely account for a majority of patients in the study being prescribed SSRIs. These may include concerns about the seizure risk with bupropion, particularly at higher dosages,^19^ and greater risk of adverse effects with bupropion in the elderly.^20^ Other possible reasons may include the greater toxicity of bupropion in overdose situations, such as in adolescents,^21^ and some evidence of greater acute improvement using an SSRI for suicidal ideation when present in those with depression.^22^ Given these trade-offs, clinicians should engage patients in a shared decision-making conversation about their choice of antidepressant treatment, taking into consideration the above potential for adverse effects from bupropion along with the potential benefits in terms of weight loss.

This study has important limitations. First, our study had a relatively small sample size and needs to be replicated in larger samples with 5-year follow-up. We found that the relative benefit of bupropion versus SSRIs was not statistically significant at 5 years, although the point estimates suggested a significant weight loss benefit of bupropion could be observed in a larger cohort. A related limitation is that we only considered antidepressant medications that were prescribed at baseline and did not have the resources to consider whether the duration or dose of antidepressant treatment after surgery may significantly influence the weight trajectory. It is also common for patients to switch antidepressant medications, and our study did not measure changes in antidepressant medications after surgery. Future research should investigate the time-varying influence of exposure to different antidepressant medications as a potential contributor to weight regain and long-term weight loss maintenance after bariatric surgery. Our sample sizes also were not large enough to permit comparisons of NDRI and SNRI medications.

An additional limitation was that patients were not randomized to bariatric surgery or to the specific antidepressant classes examined in this study, so unobserved confounding may persist, and causality cannot be inferred from this study. Surgical and nonsurgical patients were matched on several covariates that improved balance in those covariates, but several other covariates not included in the match remained imbalanced. Finally, this is a cohort of Veterans who are older and predominantly male, so results may not generalize to non-Veteran cohorts that often comprise 30–40-year-old women.

In conclusion, we found a significant association between the use of the antidepressant bupropion and superior 1-to-2 year weight loss among patients with severe obesity undergoing either gastric bypass or sleeve gastrectomy, which are the 2 most common bariatric procedures. Specifically, we found that bupropion was associated with greater weight loss than SSRIs and that SNRIs and SSRIs had similar weight loss after bariatric surgery. Therefore, we have assumed that bupropion is likely to achieve superior weight loss to SNRIs, although this assumption should be tested in larger studies. Our findings are supported by prior research on antidepressants and longitudinal weight change and suggest that bupropion should be considered the first-line antidepressant of choice for patients undergoing bariatric surgery.

## Supplementary Material



## References

[1] Arterburn DE Telem DA Kushner RF. Benefits and risks of bariatric surgery in adults: a review. JAMA. 2020;324:879–887. 32870301 10.1001/jama.2020.12567

[2] Courcoulas AP King WC Belle SH. Seven-year weight trajectories and health outcomes in the Longitudinal Assessment of Bariatric Surgery (LABS) study. JAMA Surg. 2018;153:427–434. 29214306 10.1001/jamasurg.2017.5025 PMC6584318

[3] Fisher D Coleman KJ Arterburn DE. Mental illness in bariatric surgery: a cohort study from the PORTAL network. Obesity (Silver Spring). 2017;25:850–856. 28440047 10.1002/oby.21814

[4] Susmallian S Nikiforova I Azoulai S. Outcomes of bariatric surgery in patients with depression disorders. PLoS One. 2019;14:e0221576. 31454382 10.1371/journal.pone.0221576 PMC6711535

[5] Müller M Nett PC Borbély YM. Mental illness has a negative impact on weight loss in bariatric patients: a 4-year follow-up. J Gastrointest Surg. 2019;23:232–238. 30091038 10.1007/s11605-018-3903-x

[6] Pedro J Neves JS Ferreira MJ; AMTCO Group. Impact of depression on weight variation after bariatric surgery: a three-year observational study. Obes Facts. 2020;13:213–220. 32229734 10.1159/000506404 PMC7250340

[7] Roerig JL Steffen KJ Zimmerman C. A comparison of duloxetine plasma levels in postbariatric surgery patients versus matched nonsurgical control subjects. J Clin Psychopharmacol. 2013;33:479–484. 23771193 10.1097/JCP.0b013e3182905ffb

[8] Arterburn D Sofer T Boudreau DM. Long-term weight change after initiating second-generation antidepressants. J Clin Med. 2016;5:E48. 10.3390/jcm5040048 PMC4850471 27089374

[9] Love RJ Love AS Bower S. Impact of antidepressant use on gastric bypass surgery patients’ weight loss and health-related quality-of-life outcomes. Psychosomatics. 2008;49:478–486. 19122124 10.1176/appi.psy.49.6.478

[10] Malone M Alger-Mayer SA Polimeni JM. Antidepressant drug therapy does not affect weight loss one year after gastric bypass surgery. Obes Surg. 2011;21:1721–1723. 21234700 10.1007/s11695-010-0351-4

[11] Plaeke P Van Den Eede F Gys B. Postoperative continuation of antidepressant therapy is associated with reduced short-term weight loss following Roux-en-Y gastric bypass surgery. Langenbecks Arch Surg. 2019;404:621–631. 30969361 10.1007/s00423-019-01784-z

[12] Hawkins M Leung SE Lee A. Psychiatric medication use and weight outcomes one year after bariatric surgery. Psychosomatics. 2020;61:56–63. 31806241 10.1016/j.psym.2019.10.009

[13] Kennedy EH Taylor JM Schaubel DE. The effect of salvage therapy on survival in a longitudinal study with treatment by indication. Stat Med. 2010;29:2569–2580. 20809480 10.1002/sim.4017 PMC2962719

[14] Li YP Propert KJ Rosenbaum PR. Balanced risk set matching. J Am Stat Assoc. 2001;96:870–882.

[15] Lu B. Propensity score matching with time-dependent covariates. Biometrics. 2005;61:721–728. 16135023 10.1111/j.1541-0420.2005.00356.x

[16] Qaseem A Snow V Denberg TD; Clinical Efficacy Assessment Subcommittee of American College of Physicians. Using second-generation antidepressants to treat depressive disorders: a clinical practice guideline from the American College of Physicians. Ann Intern Med. 2008;149:725–733. 19017591 10.7326/0003-4819-149-10-200811180-00007

[17] Gartlehner G Gaynes BN Hansen RA. Comparative benefits and harms of second-generation antidepressants: background paper for the American College of Physicians. Ann Intern Med. 2008;149:734–750. 19017592 10.7326/0003-4819-149-10-200811180-00008

[18] Blumenthal SR Castro VM Clements CC. An electronic health records study of long-term weight gain following antidepressant use. JAMA Psychiatry. 2014;71:889–896. 24898363 10.1001/jamapsychiatry.2014.414 PMC9980723

[19] Carvalho AF Sharma MS Brunoni AR. The safety, tolerability and risks associated with the use of newer generation antidepressant drugs: a critical review of the literature. Psychother Psychosom. 2016;85:270–288. 27508501 10.1159/000447034

[20] Sobieraj DM Martinez BK Hernandez AV. Adverse effects of pharmacologic treatments of major depression in older adults. J Am Geriatr Soc. 2019;67:1571–1581. 31140587 10.1111/jgs.15966

[21] Overberg A Morton S Wagner E. Toxicity of bupropion overdose compared with selective serotonin reuptake inhibitors. Pediatrics. 2019;144:e20183295. 31278211 10.1542/peds.2018-3295

[22] Grunebaum MF Ellis SP Duan N. Pilot randomized clinical trial of an SSRI vs bupropion: effects on suicidal behavior, ideation, and mood in major depression. Neuropsychopharmacology. 2012;37:697–706. 21993207 10.1038/npp.2011.247 PMC3260969

