# Supplementary material for: Does Long-Term Post-Bariatric Weight Change Differ Across Antidepressants?
Source: Ann Surg Open. 2022 Jan 10;3(1):e114. doi: 10.1097/AS9.0000000000000114 (PMC10013150; doi:10.1097/AS9.0000000000000114)
Supplement: Supplementary file 1 [file as9-3-e114b-s001.pdf]

eFigure 1. Flow of Matched Bariatric Surgical Patients and Non-Surgical Patients from 10/1/2000 to 9/30/2016

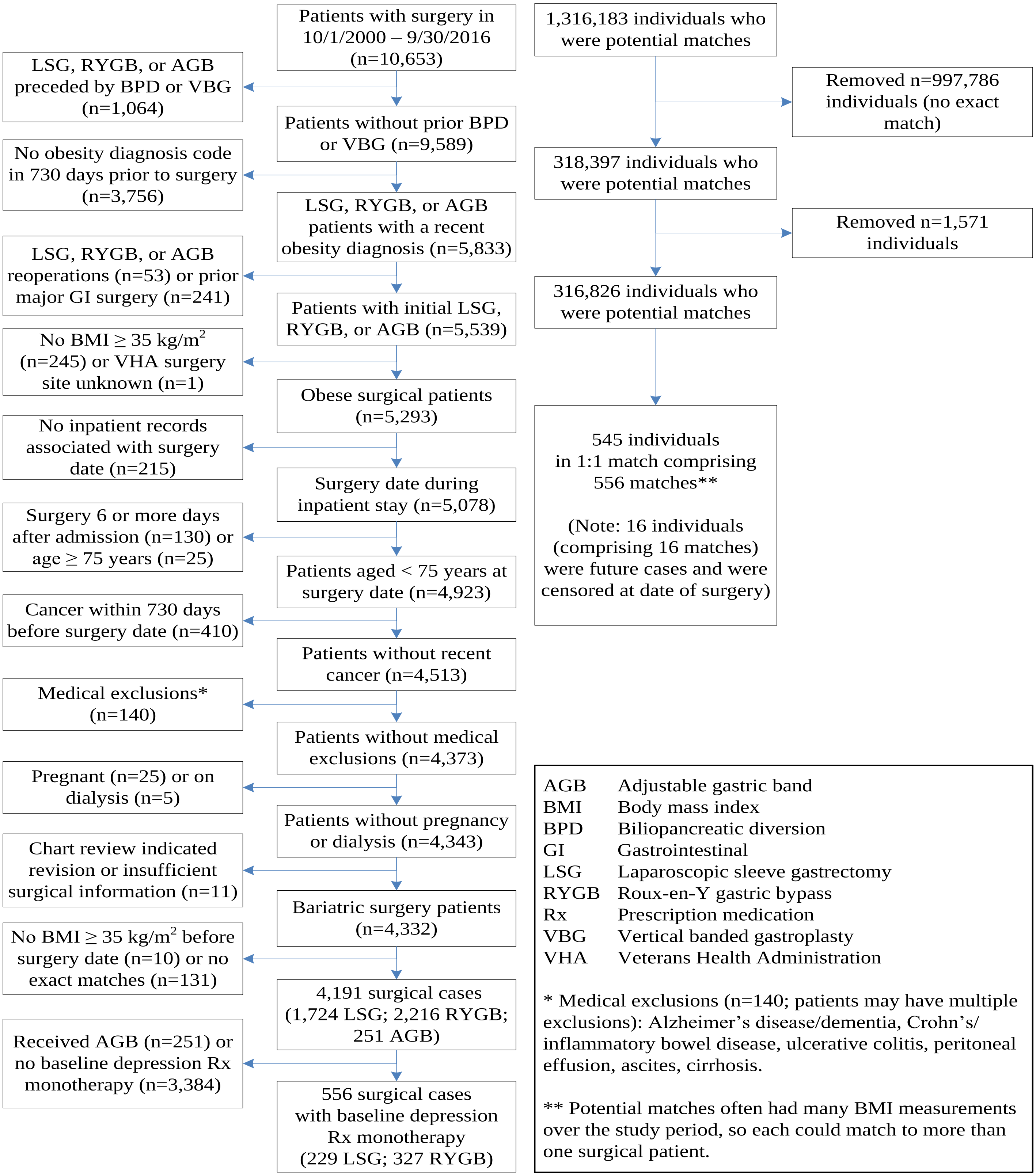

AGB	Adjustable gastric band
BMI	Body mass index
BPD	Biliopancreatic diversion
GI	Gastrointestinal
LSG	Laparoscopic sleeve gastrectomy
RYGB	Roux-en-Y gastric bypass
Rx	Prescription medication
VBG	Vertical banded gastroplasty
VHA	Veterans Health Administration

* Medical exclusions (n=140; patients may have multiple exclusions): Alzheimer's disease/dementia, Crohn's/inflammatory bowel disease, ulcerative colitis, peritoneal effusion, ascites, cirrhosis.

** Potential matches often had many BMI measurements over the study period, so each could match to more than one surgical patient.

eTable 1. Specific Medications Included in Each Antidepressant Class

SSRI	*SNRI*	*NDRI*
citalopram	desvenlafaxine	bupropion
escitalopram	duloxetine	
fluoxetine	Levomilnacipran	
fluvoxamine	milnacipran	
paroxetine	venlafaxine	
sertraline	vilazodone	

eTable 2. Mean number of weights and follow-up, by surgical type and antidepressant class

		LSG				RYGB			
		Nonsurgical control		Surgical case		Nonsurgical control		Surgical case	
Antidepressant(s)	Post-surgery year	# person-dates	Mean # weights per person-date	# person-dates	Mean # weights per person-date	# person-dates	Mean # weights per person-date	# person-dates	Mean # weights per person-date
NDRIs only	Year 0	28	5.6	28	7.8	27	6.9	29	9.4
	Year 1	25	5.0	28	6.7	27	6.1	29	7.8
	Year 2	23	5.2	27	5.8	27	8.6	29	6.4
	Year 3	22	5.5	27	5.9	27	6.8	28	6.9
	Year 4	22	5.4	27	5.4	21	5.9	27	6.2
	Year 5	21	4.5	21	5.8	21	7.0	23	6.0
SNRIs only	Year 0	39	8.2	38	9.2	31	8.1	32	9.4
	Year 1	38	8.2	39	7.5	30	8.4	31	7.1
	Year 2	35	6.5	39	5.3	30	8.4	31	6.5
	Year 3	35	6.4	38	5.1	30	7.0	27	6.1
	Year 4	26	4.9	27	5.3	24	7.9	23	5.5
	Year 5	17	5.6	17	5.8	16	10.2	20	3.8
SSRIs only	Year 0	158	6.8	160	8.8	260	6.6	265	8.9
	Year 1	155	6.0	159	6.4	261	6.6	261	7.4
	Year 2	156	5.8	153	5.7	250	6.4	259	7.1
	Year 3	147	6.1	152	5.7	238	6.6	252	6.1
	Year 4	117	6.5	127	5.3	219	6.1	231	6.2
	Year 5	92	6.1	98	5.4	204	6.0	215	5.9
